# Supplementary material for: Infralimbic medial prefrontal cortex signalling to calbindin 1 positive neurons in posterior basolateral amygdala suppresses anxiety- and depression-like behaviours
Source: Nat Commun. 2022 Sep 17;13:5462. doi: 10.1038/s41467-022-33139-6 (PMC9482654; doi:10.1038/s41467-022-33139-6)
Supplement: Supplementary file 1 — Supplementary Information [file 41467_2022_33139_MOESM1_ESM.pdf]

sFigure 1

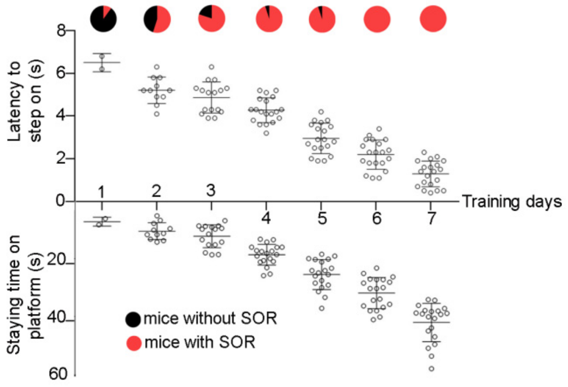

sFigure 1. Mice gradually learn to associate the negative CS tones with foot shocks. The step-on latency was gradually decreased and the staying time on the platform increased along with FC training. Red and black represent the mice with and without stepping-on response (SOR), respectively. 10 trials a day (except 10-20 trials on the first day). n = 20 mice per group. Data were presented as mean ± SEM. Source data are provided as a Source Data file.

sFigure 2

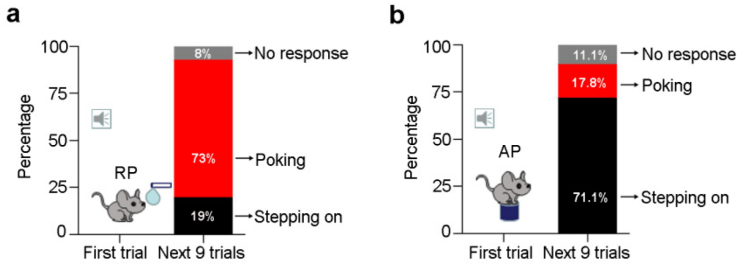

sFigure 2. The first choice is positively correlated with the subsequent 9 choices during the ambiguous tone probing. For both reward- preferred (RP, **a**) and aversion-preferred (AP, **b**) mice, the choice proportion was calculated in the next 9 trials in response to the ambiguous tone. Source data are provided as a Source Data file.

sFigure 3

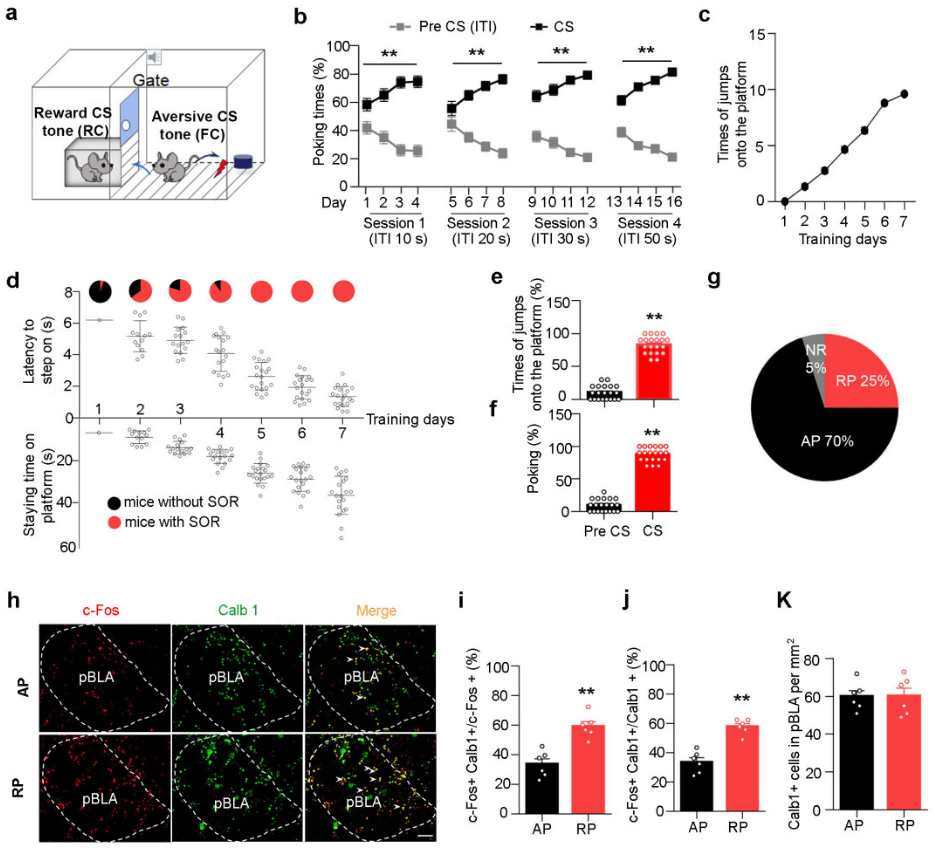

sFigure 3. The pBLA^{Calb1} neurons are robustly activated in reward-preferred mice in response to ambiguity. (**a**) Schematic of a go/go paradigm, in which the positive CS tones (2000 Hz, linked to social reward) and negative CS tones (9000 Hz, linked to foot shocks) were counterbalanced across animals. (**b**) The mice learnt to associate the positive CS tones with social reward during RC training. n = 20 mice per group. Repeated measures two-way ANOVA, [Session 1]: $F(3, 57) = 16.08$, $P<0.0001$; [Session 2]: $F(3, 57)=24.01$, $P<0.0001$; [Session 3]: $F(3, 57) = 20.49$, $P<0.0001$; [Session 4]: $F(3, 57) = 35.54$, $P<0.0001$, Tukey's multiple comparisons test, $*P < 0.01$ vs Pre CS (ITI). (**c**) The mice learnt to associate the negative CS tones with the foot shocks. 10 trials a day (except 10-20 trials on the first day). n = 20 mice per group. (**d**) Mice experienced FC training showed fewer latency and spent more time staying on the platform. n = 20 mice per group. (**e, f**) Mice experienced both RC and FC training showed correct discrimination behaviours in FC test (**e**) and RC test (**f**) in response to the CS tones, n = 20 mice per group. Two-sided paired *t* test, [Times of jumps onto the

platform%]: t = 16.46, df = 19, P<0.0001; [poking%]: t =26.39, df =19, P<0.0001. **P < 0.01 vs Pre CS. (**g**) Proportion of reward-preferred (RP), aversion-preferred (AP) and no response (NR) mice experienced the go/go paradigm. (**h-j**) The number of c-Fos (red) and Calb1 (green) co-stained neurons increased in the pBLA of RP group compared with AP group (**h-j**) with similar total Calb1-neuron numbers (**h**, **k**). Scale bar, 100 μm. n = 6 mice per group. Two-sided unpaired t test, t =5.283, df =10, P<0.0004 (**i**); t =6.641, df =10, P<0.0001(**j**), t = 0.06591, df = 10, P= 0.9487 (**k**). ** P<0.01 vs AP. Data were presented as mean ± SEM. Source data are provided as a Source Data file.

sFigure 4

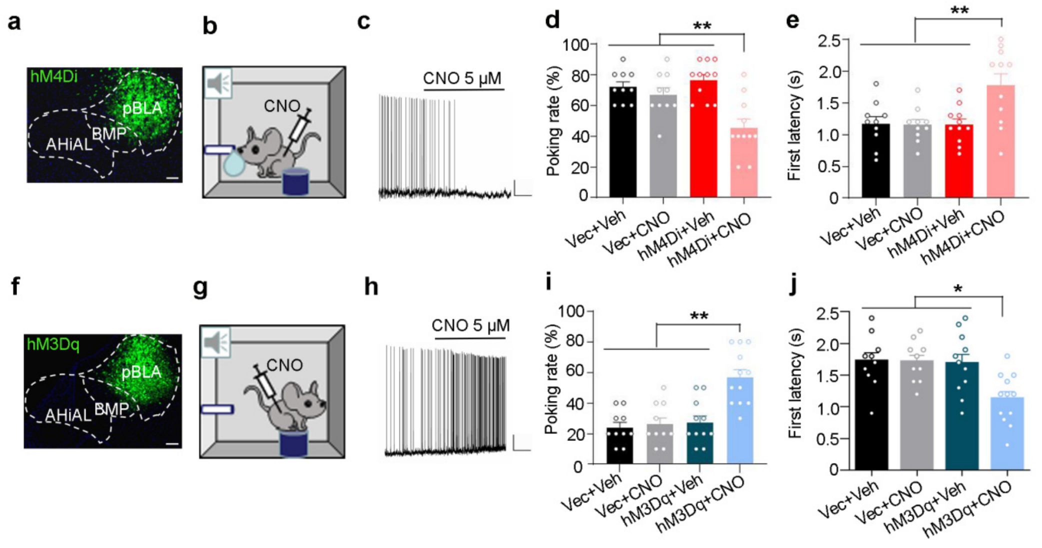

sFigure 4. pBLA neurons govern reward generalization for ambiguous cue. (**a, f**) Representative confocal images of hM4Di (**a**) and hM3Dq (**f**) expression in the pBLA. Scale bar, 100 μm. (**b, g**) Schematic of chemogenetics stimulations on hM4Di-HRG and hM3Dq-LRG mice in the presence of novel tones (ambiguous tones). (**c, h**) Representative trace recorded in current-clamp mode from pBLA neurons that expressed hM4Di or hM3Dq in the presence of CNO. Scale bars = 3 seconds, 20 mV. (**d, e**) Inhibition of pBLA decreased the poking rate (**d**) and prolonged the first poking latency (**e**) of hM4Di-HRG mice. n=10 (Vec+Veh, Vec+CNO) or 11 (hM4Di+Veh, hM4Di+CNO) mice. One-way ANOVA, $F(3,38) = 10.12$, $P = 0.0070$ (Poking rate) or $F(3,38) = 6.196$, $P = 0.0062$ (First latency). Tukey's multiple comparisons test, $^{**}P < 0.01$. (**i, j**) Activation of pBLA increased the poking rate (**i**) and shortened first poking latency (**j**) of hM3Dq-LRG mice. n=10 (Vec+Veh, Vec+CNO), 11 (hM3Dq+Veh) or 12 (hM3Dq+CNO) mice. One-way ANOVA, $F(3,39) = 13.21$, $P < 0.0001$ (Poking rate) or $F(3, 39) = 5.728$, $P = 0.0024$ (First latency). Tukey's multiple comparisons test, $^{*}P < 0.05$, $^{**}P < 0.01$. Data were presented as mean ± SEM. Source data are provided as a Source Data file.

sFigure 5

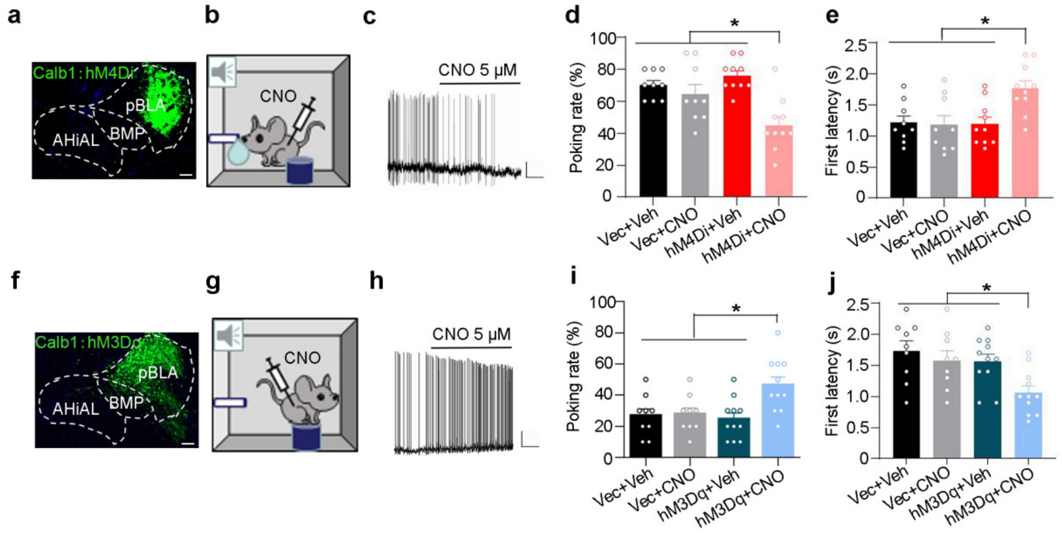

sFigure 5. pBLA^{Calb1} neurons govern reward generalization for ambiguous cue. (**a, f**) Representative confocal images of hM4Di **(a)** and hM3Dq **(f)** expression in the pBLA^{Calb1} neurons. Scale bar, 100 µm. (**b, g**) Schematic of chemogenetics stimulations on hM4Di-Calb1-HRG and hM3Dq-Calb1-LRG mice in the presence of novel tones (ambiguous tones). (**c, h**) Representative trace recorded in current-clamp mode from pBLA^{Calb1} neurons that expressed hM4Di or hM3Dq in the presence of CNO. Scale bars=3 seconds, 20 mV. (**d, e**) Inhibition of pBLA^{Calb1} neurons decreased the poking rate (**d**) and prolonged the first poking latency (**e**) of hM4Di-Calb1-HRG mice. n=9 (Vec+Veh, Vec+CNO) or 10 (hM4Di+Veh, hM4Di+CNO) mice. One-way ANOVA, $F(3,34) = 9.349$, $P = 0.0214$ (Poking rate) or $F(3,34) = 5.430$, $P = 0.0111$ (First latency). Tukey's multiple comparisons test, $^{*}P < 0.05$. (**i, j**) Activation of pBLA^{Calb1} neurons increased the poking rate (**i**) and shortened the first poking latency (**j**) of hM3Dq-Calb1-LRG mice. n=9 (Vec+Veh, Vec+CNO) or 11 (hM3Dq+Veh, hM3Dq+CNO) mice. One-way ANOVA, $F(3,36) = 5.429$, $P = 0.0035$ (Poking rate) or $F(3,36) = 4.917$, $P = 0.0489$ (First latency). Tukey's multiple comparisons test, $^{*}P < 0.05$. Data were presented as mean ± SEM. Source data are provided as a Source Data file.

sFigure 6

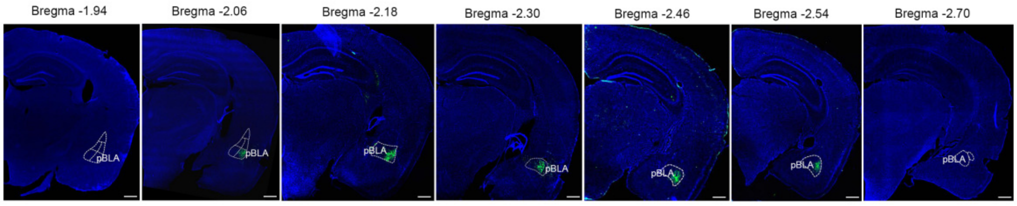

sFigure 6. Representative images of ChR2 expression in the pBLA. Most viruses have been confined to the pBLA. Scale bar, 500 μm. Experiments were successfully replicated at least three independent times.

sFigure 7

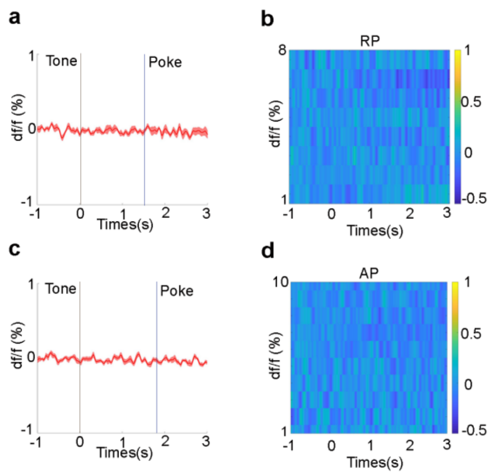

sFigure 7. No significant fluctuation of photometry in IL–pBLA–eGFP neurons. (**a, c**) Average photometry recordings from IL neurons projecting to the pBLA (IL–pBLA neurons) in the eGFP–RP mice (**a**) and eGFP–AP mice (**c**) during go/go paradigm in the presence of intermediate tones. (**b, d**) Heat maps of normalized Ca^{2+} activity in IL–pBLA neurons when the intermediate tones were present, as shown in **a, c**.

sFigure 8

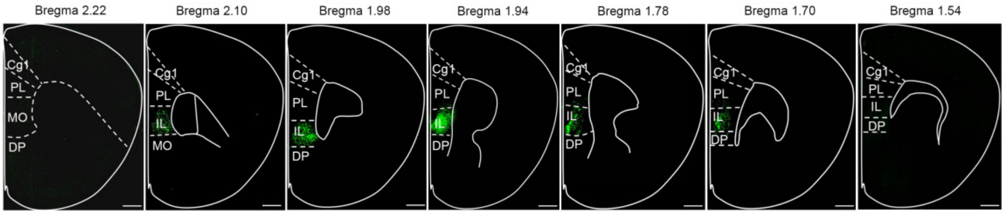

sFigure 8. Representative images of ChR2 expression in the IL. The expression of ChR2 was confined to the IL. Scale bar, 500 μm. Experiments were successfully replicated at least three independent times.

sFigure 9

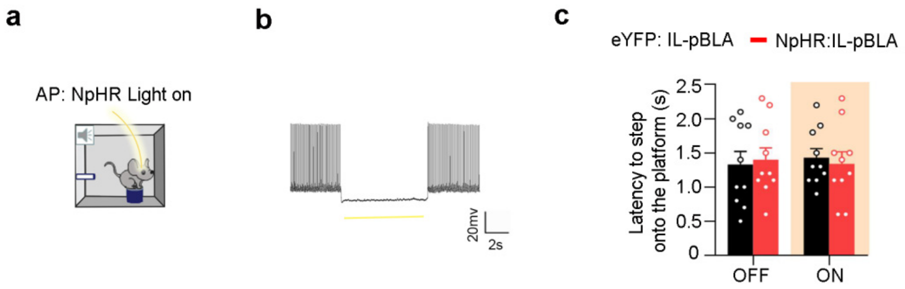

sFigure 9. Photoinhibiting IL–pBLA inputs had no effects on aversive preference in AP mice. (**a**) Schematic showing light illuminating epoch when the intermediate tone was delivered during go/go paradigm. (**b**) Yellow light illumination blocked evoked spiking of NpHR cells. (**c**) Inhibiting IL–pBLA inputs had no effect on the latency to step onto the platform in NpHR-AP mice. Two-way ANOVA group × epoch interaction; [Latency to step onto the platform] $F(1,36) = 0.2240$, $P = 0.6389$, Bonferroni post hoc analysis. n = 10 mice per group. Data were presented as mean ± SEM. Source data are provided as a Source Data file.

sFigure 10

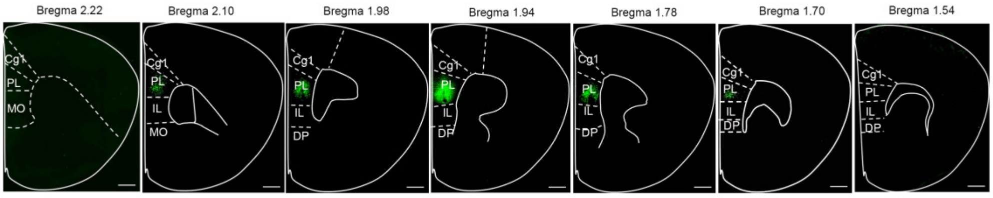

sFigure 10. Representative images of ChR2 expression in the PL. The expression of ChR2 was confined to the PL. Scale bar, 500 μm. Experiments were successfully replicated at least three independent times.

sFigure 11

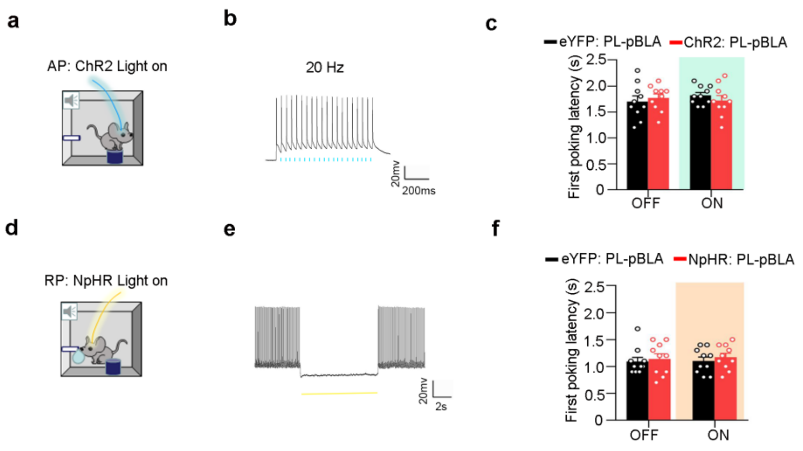

sFigure 11. PL–pBLA inputs has no effects on reward preference for the ambiguity. (**a, d**) Schematic showing light illuminating epoch when the intermediate tone was delivered during go/go paradigm. (**b, e**) Brief blue light pulses at 20 Hz precisely activated ChR2 cells (b), while yellow light illumination blocked evoked spiking of NpHR cells (e), in the pBLA. (**c, f**) Activating and inhibiting PL–pBLA inputs did not significantly change the first poking latency of ChR2-AP and NpHR-RP mice. Two-way ANOVA group × epoch interaction; first poking latency [ChR2-AP] $F(1,36) = 0.9731$, $P = 0.3305$, Bonferroni post hoc analysis; [NpHR-RP] $F(1,36) = 0.01569$, $P = 0.9010$, Bonferroni post hoc analysis. n = 10 mice per group. Data were presented as mean ± SEM. Source data are provided as a Source Data file.

sFigure 12

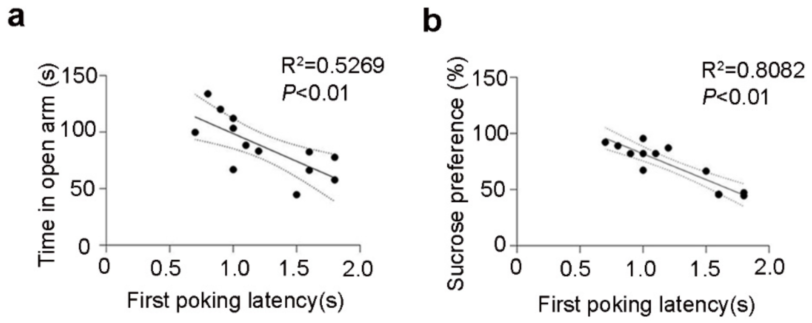

sFigure 12. Anxiety- and depression-like behaviours are negatively correlated with reward generalization for the ambiguity. (**a**) Correlation between time spent in the open arms in EPM and the first poking latency in response to intermediate tones. (**b**) Correlation between sucrose preference in SPT and the first poking latency in response to intermediate tones. n=13 mice per group. No adjustments were made. Pearson correlation coefficients were calculated. Source data are provided as a Source Data file.

sFigure 13

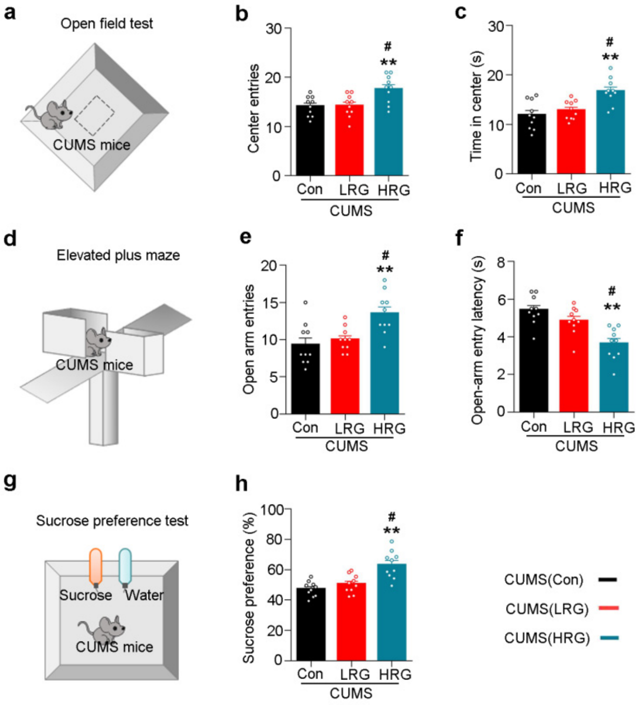

sFigure 13. High reward generalization corelates better with the resistance to CUMS than the negatives. (**a, d, g**) Schematic of open field test (OFT, **a**), elevated plus maze (EPM, **d**) and sucrose preference test (SPT, **g**). HRG-CUMS mice showed less anxiety- and depression-like behaviours, evidenced by greater center entries (**b**) and time staying in the center (**c**) in OFT, more open-arm exploration (**e**) and shorter open-arm entry latency (**f**) in EPM, and higher sucrose preference in SPT (**h**), while the suppression on anxiety- and depression-like behaviours cannot be detected in LRG-CUMS mice. One-way ANOVA, n=10 mice per group. In OFT: [Center entries] F(2,27) = 6.920, *P* =0.0037; [Time in center] F(2,27) = 10.23, *P* =0.0005. In EPM: [Open arm entries] F(2,27) = 8.206, *P* =0.0016; [Open-arm entry latency] F(2,27) = 13.43, *P* <0.0001. In SPT: F(2,27) =14.87, *P* <0.0001. Bonferroni post hoc analysis, $^{**}P < 0.01$ vs Con, # $P < 0.05$ vs LRG. Data were presented as mean ± SEM. Source data are provided as a Source Data file.

sFigure 14

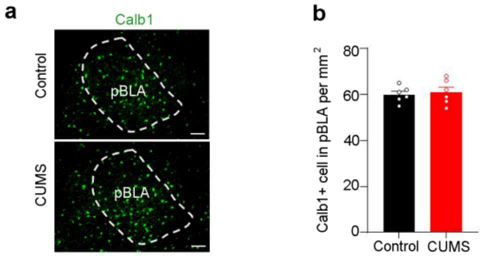

sFigure 14. There is no Calb1 neuron loss in both Control and CUMS mice.

(**a**) Representative images of Calb1 staining in the pBLA. Scale bar, 100 µm. (**b**) No difference of Calb1+ cell numbers was detected between Control and CUMS mice. n = 6 mice per group. Unpaired *t* test, t =0.3835, df =10, P=0.7094. Data were presented as mean ± SEM. Source data are provided as a Source Data file.

sTable 1

REAGENT or RESOURCE	SOURCE	IDENTIFIER
Virus Strains		
HSV-EGFP	BrainVTA	Cat# H01001
rAAV2/R-hSyn-Cre-WPRE-hGH-PA	BrainVTA	Cat# PT-0136
rAAV2/9-CaMKIIa-DIO-GCaMP6f-EGFP	BrainVTA	Cat# PT-110-3-1
rAAV2/9-CaMKIIa-DIO-EGFP	BrainVTA	Cat# PT-0119
pAAV2/8-CaMKIIa-hChR2(H134R)-eYFP	OBio	N/A
pAAV2/8-CaMKIIa-eNpHR3.0-eYFP	OBio	N/A
pAAV2/8-CaMKIIa-eYFP	OBio	N/A
pAAV2/8-CaMKIIa-hM3D(Gq)-eYFP	OBio	N/A
pAAV2/8-CaMKIIa-hM4D(Gi)-eYFP	OBio	N/A
pAAV2/8-CaMKIIa-DIO-hM3D(Gq)-eYFP	OBio	N/A
pAAV2/8-CaMKIIa-DIO-hM4D(Gi)-eYFP	OBio	N/A
pAAV2/8-CaMKIIa-DIO-eYFP	OBio	N/A
pAAV2/8-CaMKIIa-DIO-hChR2(H134R)-eYFP	OBio	N/A
pAAV2/8-CaMKIIa-DIO-eNpHR3.0-eYFP	OBio	N/A
Experimental Models: Organisms/Strains		
C57BL/6 mice	Beijing Vital River Laboratory Animal Technology Co., Ltd.	N/A
Calb1-IRES2-Cre-D mice	Xiaohui Zhang lab	N/A
Ai9 mice	Xiaohui Zhang lab	N/A
Chemicals		
Clozapine N-oxide (CNO)	Sigma	Cat# C0832
DAPI	Beyotime	Cat# C1002
CTB555	BrainVTA	Cat# CTB-02

sTable 2 Antibody list

Antibodies	Host	Source	Cat#	Dilution
c-Fos	Rabbit	Synaptic Systems	226003	1:300
Calbindin D-28k	Mouse	Swant	300	1:500
Calbindin antibody	Rabbit	Abcam	ab108404	1:500
Alexa Fluor 488 donkey-anti-rabbit IgG(H+L)	Donkey	Invitrogen	A21206	1:300
Alexa Fluor 488 donkey-anti-mouse IgG(H+L)	Donkey	Invitrogen	A21202	1:300
Alexa Fluor 546 donkey-anti-rabbit IgG	Donkey	Invitrogen	A10040	1:300

sTable 3

Week	Day	Food deprivation	Water deprivation	Foreign object exposure	Cold exposure	Illumination	Restraint
1	1	24h (6:00 a.m.-6:00 a.m. on day 2)	24h (6:00 a.m.-6:00 a.m. on day 2)				
	2			1 h (5:00 a.m.-6:00 a.m.)			2 h (6:00 p.m.-8:00 p.m.)
	3				2 h (9:00 a.m.-11:00 a.m.)	12 h (6:00 p.m.-6:00 a.m. on day 4)	
	4				2 h (7:00 a.m.-9:00 a.m.)		2 h (4:00 p.m.-6:00 p.m.)
	5	24 h (6:00 a.m.-6:00 a.m. on day 6)		1 h (7:00 p.m.-8:00 p.m.)			
	6		24h (6:00 a.m.-6:00 a.m. on day 7)			12 h (7:00 a.m.-7:00 p.m.)	
	7			1 h (9:00 a.m.-10:00 a.m.)	2 h (4:00 p.m.-6:00 p.m.)		

Week	Day	Food deprivation	Water deprivation	Foreign object exposure	Cold exposure	Illumination	Restraint
2	1	24h (6:00 a.m.-6:00 a.m. on day 2)					2 h (6:00 p.m.-8:00 p.m.)
	2		24h (6:00 a.m.-6:00 a.m. on day 3)			12 h (6:00 p.m.-6:00 a.m. on day 3)	
	3			1 h (5:00 a.m.-6:00 a.m.)	2 h (9:00 a.m.-11:00 a.m.)		2 h (4:00 p.m.-6:00 p.m.)
	4	24 h (6:00 a.m.-6:00 a.m. on day 5)		1 h (7:00 p.m.-8:00 p.m.)			
	5				2 h (7:00 a.m.-9:00 a.m.)		2 h (4:00 p.m.-6:00 p.m.)
	6		24h (6:00 a.m.-6:00 a.m. on day 7)	1 h (4:00 p.m.-5:00 p.m.)			2 h (8:00 p.m.-10:00 p.m.)
	7				2 h (4:00 p.m.-6:00 p.m.)	12 h (6:00 a.m.-6:00 p.m.)	2 h (8:00 a.m.-10:00 a.m.)

Week	Day	Food deprivation	Water deprivation	Foreign object exposure	Cold exposure	Illumination	Restraint
3	1		24h (6:00 a.m.-6:00 a.m. on day 2)				2 h (6:00 p.m.- 8:00 p.m.)
	2	24h (6:00 a.m.-6:00 a.m. on day 3)		1 h (5:00 a.m.-6:00 a.m.)	2 h (4:00 p.m.-6:00 p.m.)		
	3					12 h (6:00 p.m.-6:00 a.m. on day 4)	2 h (4:00 p.m.-6:00 p.m.)
	4		24h (6:00 a.m.-6:00 a.m. on day 5)				2 h (4:00 p.m.-6:00 p.m.)
	5			1 h (7:00 a.m.-8:00 a.m.)	2 h (4:00 p.m.-6:00 p.m.)	12 h (6:00 a.m.-6:00 p.m.)	
	6	24h (6:00 a.m.-6:00 a.m. on day 7)			2 h (9:00 a.m.-11:00 a.m.)		2 h (3:00 p.m.-5:00 p.m.)
	7			1 h (1:00 p.m.-2:00 p.m.)	2 h (4:00 p.m.-6:00 p.m.)		2 h (8:00 a.m.- 10:00 a.m.)

sTable 3: Experimental Stressors and Scheduling for Chronic Unpredictable Mild Stress (CUMS). Mice were exposed to a variety of mild unexpectable stressors for 6 weeks, including water and/or food deprivation, foreign object exposure, restraint, cold exposure, and reversal of the light/dark cycle. The procedures were repeated from week 1 after first 3 weeks. Some stressors were interrupted or modified when they occurred at particular points in the sucrose-preference test schedule.
